# Combination of phenotype and polygenic risk score in breast cancer risk evaluation in the Spanish population: a case –control study

**DOI:** 10.1186/s12885-020-07584-9

**Published:** 2020-11-10

**Authors:** J. C. Triviño, A. Ceba, E. Rubio-Solsona, D. Serra, I. Sanchez-Guiu, G. Ribas, R. Rosa, M. Cabo, L. Bernad, G. Pita, A. Gonzalez-Neira, G. Legarda, J. L. Diaz, A. García-Vigara, A. Martínez-Aspas, M. Escrig, B. Bermejo, P. Eroles, J. Ibáñez, D. Salas, A. Julve, A. Cano, A. Lluch, R. Miñambres, J. Benitez

**Affiliations:** 1 Sistemas Genómicos, Ronda Guillermo Marconi 6, Parque Tecnológico, 46980 Paterna, Valencia Spain; 2 Spanish National Genotyping Center (CEGEN), Madrid, Spain; 3 Human Cancer Genetics Programme, Spanish National Cancer Center (CNIO), Melchor Fernandez Almagro 3, 28029 Madrid, Spain; 4 Obstetrics and Gynecology Service, Hospital Clínico Universitario – INCLIVA, Av Blasco Ibáñez 17, 46010 Valencia, Spain; 5 Department of Hematology and Medical Oncology, Hospital Clínico Universitario de Valencia, University of Valencia, INCLIVA Biomedical Research Institute, Valencia, Spain; 6 Biomedical Research Centre Network in Cancer (CIBERONC), Madrid, Spain; 7 General Directorate Public Health, Valencian Community, Valencia, Spain; 8 Valencia Cancer and Public Health Area, FISABIO - Public Health, Valencia, Spain; 9 Consortium for Biomedical Research in Epidemiology and Public Health (CIBER Epidemiología y Salud Pública, CIBERESP), Valencia, Spain; 10 Radiology Service, Hospital Clínico Universitario – INCLIVA, Av Blasco Ibáñez 17, 46010 Valencia, Spain

**Keywords:** Polygenic risk score, Predictive test, Identification of high risk women, Risk algorithms

## Abstract

**Background:**

In recent years, the identification of genetic and phenotypic biomarkers of cancer for prevention, early diagnosis and patient stratification has been a main objective of research in the field. Different multivariable models that use biomarkers have been proposed for the evaluation of individual risk of developing breast cancer.

**Methods:**

This is a case control study based on a population-based cohort. We describe and evaluate a multivariable model that incorporates 92 Single-nucleotide polymorphisms (SNPs) (Supplementary Table S1) and five different phenotypic variables and which was employed in a Spanish population of 642 healthy women and 455 breast cancer patients.

**Results:**

Our model allowed us to stratify two groups: high and low risk of developing breast cancer. The 9th decile included 1% of controls vs 9% of cases, with an odds ratio (OR) of 12.9 and a *p*-value of 3.43E-07. The first decile presented an inverse proportion: 1% of cases and 9% of controls, with an OR of 0.097 and a p-value of 1.86E-08.

**Conclusions:**

These results indicate the capacity of our multivariable model to stratify women according to their risk of developing breast cancer. The major limitation of our analysis is the small cohort size. However, despite the limitations, the results of our analysis provide proof of concept in a poorly studied population, and opens up the possibility of using this method in the routine screening of the Spanish population.

**Supplementary Information:**

The online version contains supplementary material available at 10.1186/s12885-020-07584-9.

**Supplementary Information:**

The online version contains supplementary material available at 10.1186/s12885-020-07584-9.

## Background

The prevention and early diagnosis of breast cancer is one of the main objectives of cancer research. There are different models to estimate cancer risk based on genetic or non-genetic factors; that is, a high or moderate predisposition [1, 2]. In recent years, the extensive use of genome-wide association studies (GWAS) has led to the identification of low-susceptibility alleles (SNPs). These SNPs are usually combined in a polygenic risk score (PRS), which, in combination with non-genetic factors, reflects the risk of developing breast cancer [3]. We recently described a low-susceptibility SNP polygenic risk score of 76 for breast cancer that allows the general population to be stratified. According to this score, women at a low and high risk of developing breast cancer presented 0.5 and 2.5-fold increased risks, respectively, relative to women in the middle quintile [4]. Previous studies have shown that breast density, familial antecedents and PRS models composed of 77 [5], 83 [6] or, more recently, 313 SNPs [7] determine women at risk. The combination of phenotype and PRS increases the likelihood of identifying women at risk who require personalized follow-up, particularly when an individual exceeds the risk threshold.

Although there are previous studies in Caucasian populations, this is the first to combine a PRS of 92 SNPs with other risk factors, such as mammographic density (MD), reproductive factors, and family history, in a Spanish population of 1097 women. The main objective was to analyze the usefulness of this approach in our population using a multivariable logistic method based on the combination of these variables.

## Methods

### Study design: description of cohorts

The present study was submitted to and approved by the Clinical Research Ethics Committee (CEIC) of the Hospital Clínico Universitario de Valencia (Spain) - September 29th, 2016 (2016/169) and July 13th, 2018 (2018/139) - and was conducted in compliance with the Helsinki Declaration.

This is case control study compiling full genotyping and phenotypic data for a cohort recruited between January 2017 and December 2018 from two sources: Hospital Clínico Universitario de Valencia and Valencian Community Screening Programme (General Directorate Public Health), both in the Autonomous Community of Valencia (on the Mediterranean Coast). A total of 867 healthy women and 640 breast cancer patients were recruited, with ages in the range of 30–70. Patients had developed breast cancer in a maximum period of 5 years prior to data collection, while controls were women who had not developed breast cancer during the same period. Those that presented incomplete phenotypic data or genotyping failure were excluded from the cohort, which left 1097 participants consisting of 642 healthy women and 455 breast cancer cases.

The patient cohort was composed of 45% Luminal A, 20% Luminal B, 20% Her-2 positive and 15% Triple Negative tumors (approximate percentages).

### Data collection

Clinical information was collected for all subjects at recruitment: family history of breast cancer, date of birth, age, age at menarche, age at menopause, age at first pregnancy, and mammographic density (MD). Breast density was assessed from craniocaudal and mediolateral oblique mammographic projections by an experienced radiologist with more than 10 years of experience. The radiologist used the image viewer system (DICOM, from General Electric GIMD company), classifying MD according to Boyd’s semiquantitative scale [8].

### SNP selection and genotyping

As in our previous PRS risk analysis [4], we initially selected 76 SNPs from the European Collaborative Oncological Gene Environment Study (COGS) [9]. These SNPs were significant or showed a trend towards significance in our previous validation with Spanish samples. The correlation of the genetic variants analyzed with prediction of breast cancer risk in women of the Spanish population has already been described [4]. In brief, we analyzed the performance of our PRS using the 76 selected SNPs for breast cancer risk prediction in a Spanish case and control cohort. The initial selection was extended to 123 SNPs by including additional SNPs obtained from the OncoArray Project [10]. Of these, 28 SNPs with an OR close to 1 (0.95 < OR < 1.05) and another 3 SNPs with platform genotyping failure were removed. In this way, a total of 92 SNPs [11–16] were eventually employed for the current analysis (Online Resource 1).

The genotyping method has been described previously [4]. In short, 10 ml of peripheral blood was collected in an EDTA tube. One μg of Deoxyribonucleic acid (DNA) was used for the genotypic analysis (minimum concentration of 25 ng/μL). Genotyping was performed with the Open Array® Real-Time PCR platform (Life Technologies) using the Acufill® system and Taqman® probes. The data obtained were analyzed using Genotyper software. Samples with a call rate < 0.95 were discarded. SNPs with a genotyping rate < 0.95 and SNPs generating errors in control duplicates were also ruled out.

### Statistical analysis

Sample size was calculated with a 95% confidence level (two-tailed test), 80% statistical power, control-case ratio of 1.3 and initial prevalence of breast cancer of 12%; the total number of women necessary for results to be statistically significant was 1138, similar to our case control cohort (1097). In an initial exploratory univariable process, the case/control ratio of each risk factor was compared. During this step, the Wilcoxon-test was used with a two-sided *p*-value threshold of 0.05.

The PRS was based on a combined effect of 92 SNPs statistically associated with breast cancer. This strategy considers an independent effect of each SNP, ignoring departures from a multiplicative model [17]. The PRS was derived for each study subject using the formula:

$$ PRS=\beta 1x1+\beta 2x2+\dots +\beta kxk+\dots +\beta 92x92 $$

where *xk* is the number of risk alleles (0, 1 or2) based on the ploidy of each SNP. The *βk* weights are the ORs of the risk alleles associated with breast cancer described in Online Resource 1. This strategy has been used in other studies [5, 6]. The resulting values are normalized using the median PRS value of the control samples of the cohort.

In the phenotypic analysis, the phenotypic categories were transformed into quantitative variables using the ORs described in the *Pollan* et al. study [8], except for family history, the ORs of which were based on the *Pharoah* et al. study [18]. In addition, the age of women (age at diagnosis of patients and age at interview of controls) was grouped into five-year periods, similar to in other publications [19], which allowed the groups to be transformed into quantitative variables. The final number of cases and controls in our cohort was 455 and 642, respectively.

For the univariable analysis, logistic regression was applied to each risk factor, which has been adjusted for age and centre. The coefficients of the model were standardized using the reghelper library of R [20]. Additionally, the PRS was adjusted for the first five principal components. The interaction effect between variables was also evaluated using the likelihood ratio test (LRT). All analyses were two-sided and employed a *p*-value threshold of 0.05.

To confirm the independence of the PRS and other phenotypic risk factors, pairwise Spearman correlations of unaffected controls were evaluated.

For the multivariable study, we performed a logistic regression analysis that incorporated the statistically significant variables obtained in the previous steps, including the interaction terms. Family history and age at menarche were also included in the analyses, even though they were not significant, since they are well-known risk factors. The significance of the final model was evaluated using the Wald Test [21]. To assess the accuracy of the final multivariable model, a global Hosmer-Lemeshow goodness-of-fit test was performed using deciles [22].

To evaluate improvement in risk prediction for the different models and risk factors, the area under the curve (AUC) was evaluated [23] as a measure of discrimination between cases and control women. This calculation was performed using the pROC [24] library of R. To avoid a possible overfitting of the model, the 95% Confidence Interval (CI) of the AUC was assessed using a cross validation strategy [25]. This step was based on the calculation of AUC in 1000 permutations using a random selection of 90% of women as a training set and the remaining 10% as a test set.

Finally, women were stratified into deciles based on their final individual risk factor, obtained from the multivariable model. The ORs of extreme deciles were evaluated using logistic regression with a reference range of 40–60%.

Based on the characteristics of our cohort, the final individual risk factor proposed in this study describes the relative risk of women in the Spanish population of suffering breast cancer in a maximum period of 5 years.

## Results

### Association of phenotypic risk factors with breast cancer

Age is one of the most important risk factors of breast cancer [26]. To ensure that our analysis was not affected by any bias or confounding effect associated with this risk factor, the distribution of cases and controls was compared using the Wilcoxon test, with no significant differences being detected (*p*-value of 0.27). The median age of our cohort was 51 years old, with a range of 30 and 70 years in the extreme deciles (Table 1).

**Table 1:** Phenotypic and genotypic baseline characteristics of cases and controls in our Spanish cohort

Risk Factor	Category	Description	Number	%	Number	%	Median	SD	Median	SD	OR	OR CI 95%	***P***-value
			Controls	Controls	Cases	Cases	Control	Control	Cases	Cases			
**Age**	0	30–35 years	28	4,36	16	3,52	51	8,18	51	8,14	1,05	0.79–1.13	0,27
	1	35–40 years	58	9,03	28	6,15							
	2	40–45 years	84	13,08	63	13,85							
	3	45–50 years	138	21,5	115	25,27							
	4	50–55 years	158	24,61	86	18,9							
	5	55–60 years	113	17,6	94	20,66							
	6	60–65 years	47	7,32	37	8,13							
	7	> 65 years	16	2,49	16	3,52							
**Breast Density**	0	From 0 to 10%	99	15,42	51	11,21	2	1,2	3	1,3	1,46	1.21–1.71	1,64E-07
	1	From 11 to 25%	116	18,07	53	11,65							
	2	From 26 to 50%	185	28,82	116	25,49							
	3	From 51 to 75%	181	28,19	133	29,23							
	4	Greater than 75%	61	9,5	102	22,42							
**Age at first delivery**	0	Less than 20 years	33	5,14	23	5,05	2	1,4	2	1,46	1,15	1.02–1.31	0,03
	1	From 20 to 24 years	165	25,7	104	22,86							
	2	From 25 to 29 years	203	31,62	107	23,52							
	3	From 30 to 34 years	106	16,51	101	22,2							
	4	Greater than 34 years	56	8,72	50	10,99							
	5	Nulliparous	79	12,31	70	15,38							
**Age at menopause**	0	Less than 46 years	97	15,11	47	10,33	2	1,4	3	1,13	1,96	1.72–2.24	2,20E-16
	1	From 46 to 50 years	147	22,9	102	22,42							
	2	Greater than 50 years	110	17,13	71	15,6							
	3	Premenopause	87	13,55	212	46,59							
	4	Menstruating	201	31,31	23	4,97							
**Age at menarche**	0	Equal to or greater than 15 years	34	5,3	34	7,47	2	1,21	3	1,2	0,89	0.78–1.04	0,061
	1	14 years	115	17,91	85	18,68							
	2	13 years	178	27,73	100	21,98							
	3	12 years	140	21,81	110	24,18							
	4	Less than 12 years	175	27,26	123	27,03							
	5	Null	0	0	3	0,66							
**Family antecedents**	0	No affected relative	468	72,9	308	67,69	0	1,16	0	1,23	1,05	0.93–1.19	0,34
	1	A first-degree relative diagnosed with breast cancer at age 50 years or older	52	8,1	43	9,45							
	2	A first-degree relative diagnosed with breast cancer when younger than 50 years	25	3,89	18	3,96							
	3	1 affected second-degree relative	90	14,02	79	17,36							
	4	2 affected first-degree relatives	4	0,62	5	1,1							
	5	2 affected second-degree relatives	1	0,16	2	0,44							
	6	3 or more affected relatives	2	0,31	0	0							

The global phenotypic risk factors after comparison between cases and controls in our cohort are detailed in Table 1. Differences between cases and controls in age at menarche and familial antecedents were not statistically significant, with *p*-values of 0.061 and 0.34, respectively.

Mammographic density presented a clear, statistically significant relationship with breast cancer, with an OR of 1.46 (95% CI: 1.21–1.71) and a p-value of 1.64E-7. The main differences between controls and cases were concentrated in the extremes, with respective proportions of 15% versus 11% in the first category (MD 0–10%) and 10% versus 22% in the last category (MD > 75%).

In our cohort, a higher age at first delivery was associated with an increased risk of development of breast cancer, while age at menarche did not have a statistically significant effect, with *p*-values of 0.03 and 0.061, respectively. Age at first delivery was associated with an OR of 1.15 (95% CI: 1.02–1.31), and the most marked differences were seen with advanced maternal ages (over 34 years), with a proportion of 11% versus 8% among cases and controls, respectively. In terms of age at menarche, the OR was 0.89 (95% CI: 0.78–1.04). Another reproductive factor we have considered in this study was menopause status, which was associated with an OR of 1.96 (95% CI: 1.72–2.24) and a *p*-value <2E-16. The greatest difference between cases and controls was observed in the premenopausal category, with values of 46 and 13%, respectively. Regarding family history, cases showed a slightly stronger trend towards more breast cancer antecedents in first- and second-degree family members than controls; however, the logistic regression based on this quantitative variable was not statistically significant, with a p-value of 0.34.The interaction terms identified in our analysis as statistically significant and included in the multivariable model were age with mammary density and age with menopause status, with *p*-values of 0.004 and 2E-16, respectively. Indeed, the relation between both phenotypes and age has been the subject of study in the field of breast cancer for some time [27–29].

The discriminative power of each phenotypic risk factor was compared using ROC curve analysis generated by 10-fold cross-validation (Table 2). The results were concordant with the univariable logistic regression, where age at menarche and family history did not present significant trends and the most discriminant phenotypic variables were menopause status - with an AUC of 0.64 (95% CI: 0.58–0.70) - and mammographic density - with an AUC of 0.60 (95% CI: 0.56–0.66) (Table 2).

**Table 2:** Age-adjusted AUC for univariable and multivariable models

Model	Median AUC	95% CI AUC	***P***-value
Breast Density	0.60	0.54–0.66	2.17E-03
Age at first delivery	0.54	0.48–0.60	1.49E-01
Age at Menopause	0.64	0.58–0.70	5.40E-09
Familial Antecedents	0.52	0.47–0.58	6.45E-01
Age at Menarche	0.53	0.48–0.59	2.80E-01
PRS92	0.62	0.56–0.66	3.64E-03
Multivariable model without interactions	0.74	0.71–0.77	2.20E-16
Multivariable model with interactions	0.8	0.77–0.83	2.20E-16

### Association of PRS92 with breast cancer

**Fig. 1:**
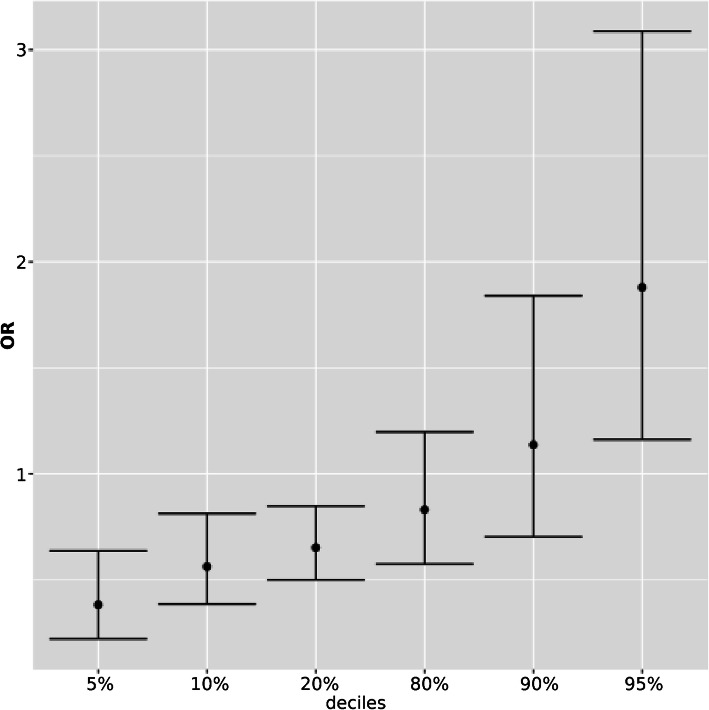
Odds ratios by decile of polygenic risk score, estimated in the Spanish population using 92 SNPs (PRS92). The PRS were converted to deciles and the 40–60% range was used as a reference. Odds ratios and 95% confidence intervals (error bars) were estimated using logistic regression

The PRS based on 92 SNPs presented an OR per 1 standard deviation (SD) of 1.41, with a 95% CI of 1.24–1.61 and a *p*-value of 6.30 × 10^− 8^. For women in the lowest quintile (5%), the PRS distribution presented an OR of 0.38 (95% CI: 0.22–0.63; *p*-value = 0.0026) with respect to women in the middle quintile (40–60%). On the other hand, the highest quintile (95%) of PRS distribution exhibited an OR of 1.87 (95% CI: 1.16 3.08; *p* = 0.036) (Fig. 1). The x-axis corresponds with the different deciles and the y-axis reflects the OR using the 40–60% range as reference. The discriminative accuracy of PRS92 was calculated using the area under the curve (AUC). PRS92 (adjusted by age) and the first five principal components presented a discriminative power of 0.62 and a 95% CI of 0.56–0.66 (Table 2). This predictive performance range was one of the most discriminant variables, along with breast mammographic density (0.60) and menopause (0.64).

### Multivariable model for breast cancer stratification

All statistically significant univariable risk factors and interaction terms were included in the final multivariable model. Age at menarche and family history were also incorporated into the model based on the scientific literature. The Spearman method did not reveal significant correlations for any other variable (data not shown).

**Fig. 2:**
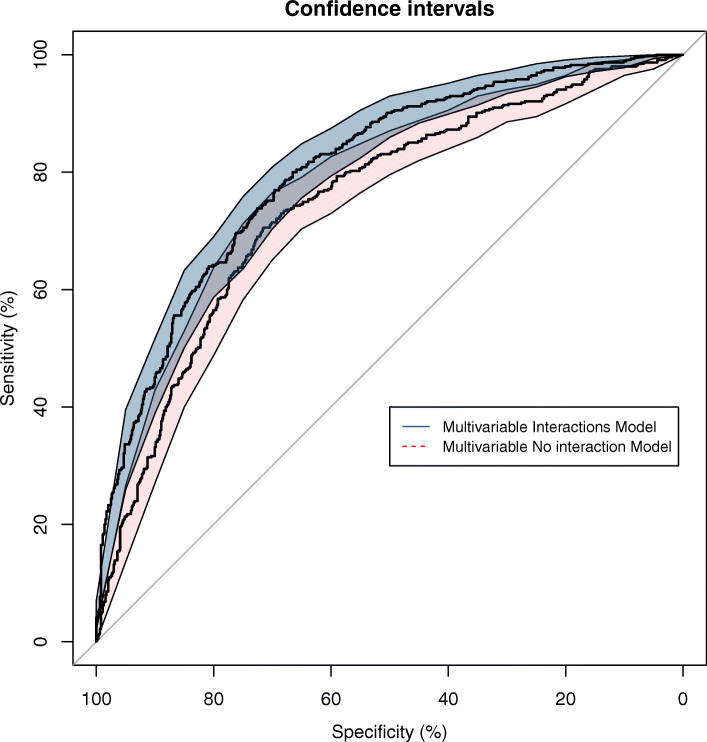
AUC-ROC of the multivariable model, with and without interaction terms (blue and pink, respectively). The AUC of the ROC curve of the final multivariable model with interaction was significantly higher than that of the model without interaction: 0.80 (95% CI: 0.77–0.83) versus 0.74 (95% CI: 0.71–0.77). The 95% confidence interval was evaluated using a bootstrap strategy

We evaluated the discriminative accuracy of the multivariable model with and without interaction terms (Fig. 2). The median AUC obtained using the interaction model was 0.80 (95% CI: 0.77–0.83), which was higher than that for the model without interactions; 0.74 (95% CI: 0.71–0.77) (Table 2). This difference was statistically significant with a *p*-value of 5.375E-09. These values are slightly higher than those observed in other previously published methods [30, 31].

**Fig. 3:**
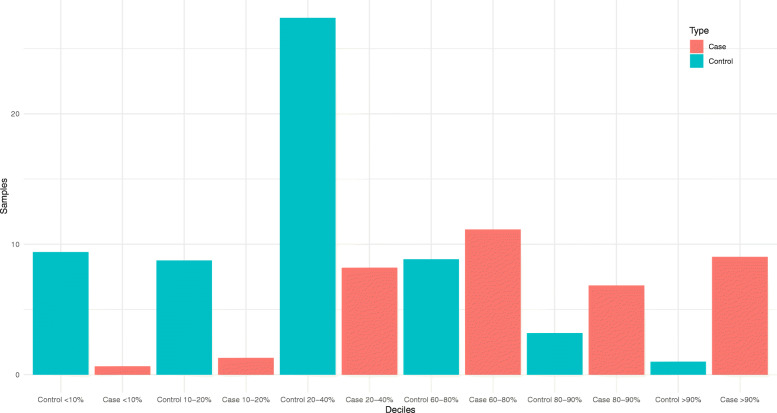
Case and control distribution using the multivariable model with interactions. The risk calculated from the model was categorized in deciles using the 40–60% range as a reference. The distribution of cases and controls are described in red and blue, respectively

We investigated how individual risk for cases and controls differed when the final multivariable model was used. Figure 3 and Table 3 show the ORs and percentages of cases and controls classified by deciles using the final risk predicted by the multivariable model with interactions. In the first decile, the OR was 0.097 (CI: 95% 0.046–0.184) with a p-value of 1.86E-08. This range contained 9% of controls versus less than 1% of cases. This trend was similar in the next decile, with 8.75 and 1.28% of controls and cases, respectively (OR: 0.29; *p* = 8.12E-07). At the other extreme, in the last decile, OR was 12.9 (CI: 95% 5.098–23.332; *p* = 3.43E-07), and the proportion of cases and control was inversed, with 9% of cases and 1% of controls. These results indicate the capacity of the multivariable model to stratify women according to the risk they run of suffering breast cancer.

**Table 3:** ORs, 95% CI and distribution of cases and controls in deciles

Deciles	OR	OR 5%	OR 95%	***P***-value	% Controls	% Cases
< 10%	0.097	0.046	0.184	1.86E-08	9.39	0.64
10–20%	0.209	0.121	0.345	8.12E-07	8.75	1.28
20–40%	0.402	0.282	0.570	1.99E-05	27.35	8.20
60–80%	1.803	1.313	2.481	2.30E-03	8.84	11.12
80–90%	3.071	2.057	4.634	5.31E-06	3.19	6.84
> 90%	12.900	5.098	23.332	3.43E-07	1.00	9.02

Results obtained using the multivariable model with interactions. The 40–60% range was selected as a reference

## Discussion

In recent years, there have been various proposals for multivariable models that stratify women who might suffer breast cancer according to their individual risk. Different biomarkers have been analyzed as possible predictors, including phenotypic and non-phenotypic markers, and environmental and genetic factors.

One approach to measuring genetic variables is the polygenic risk score (PRS). This strategy is based on variable numbers of statistically significant low penetrance variants obtained from large GWAS analyses [5, 32].

Our study was based on a relatively small cohort of women adjusted for center of origin in our univariable and multi-variable models.

Employing a specific PRS based on 92 SNPs we obtained an OR of 1.41 (1.24–1.61) that was consistent with the results of other published studies of Caucasian populations using different numbers of SNPs (from 18 to 313) [5, 32–34].

The AUC-ROC was 0.62, with a 95% CI of 0.56–0.66, which is also in line with the literature and assigns a range of 0.58 to 0.65 to European populations and one of 0.53 to 0.64 to non-European populations [35].

Regarding univariable phenotypic risk factor analysis, the most statistically significant results in terms of discriminant variables were obtained for menopause status and mammographic density, which once again is consistent with previous studies [28, 29, 36, 37]. Other reproductive factors, such as later age when giving birth for the first time and later age at menarche, have been identified as risk factors for breast cancer [38]. In our study, a significant *p*-value of 0.03 and an OR of 1.15 were identified for the former risk factor, while the latter was not found to be statistically significant (*p*-value = 0.061).

The ORs of the risk factors obtained in our cohort present differences with respect to those previously reported. The most evident concern the lack of a statistical significance of family history and age of menarche. However, the direction (positive or negative) of these well-established effects and our results are concordant. On the other hand, the magnitude of OR of mammographic density was lower than that reported in the literature. These differences may be due to the low number of women in our cohort; however, the concordance of the effect, direction and magnitude of the different ORs of our population corroborates the validity of our study as a first proof of concept in a Spanish population.

Additionally, the joint association of our PRS92 with transformed continuous phenotypic variables, such as MD, reproductive factors and family history, was examined in our Spanish population. We did not find any significant correlation between genotypic and phenotypic variables; a multiplicative model would possibly describe this in greater depth and help to improve breast cancer risk estimation.

The precision of the multivariable model increased when we added two statistically significant interaction terms associated with women’s age: menopause and mammographic density. Such interactions have previously been observed, and we detected an increase of AUC-ROC from 0.74 (95% CI: 0.71–0.77) to 0.80 (95% CI: 0.77–0.83) (Table 2), a rise that was statistically significant and offered a final value slightly higher than those of other similar multivariable studies [39].

We were able to stratify the control group within our model (Fig. 3), in which both extremes showed important differences. The last decile included 1% of controls vs 9% of cases, with an OR of 12.9 and a *p*-value 3.43E-07. In contrast, the first decile presented an inverse proportion (1% of cases and 9% of controls); in this case, the OR was 0.097, with a p-value of 1.86E-08. These results indicate the capacity of the multivariable model to stratify women according to risk of developing breast cancer.

In summary, our results indicate that using the multivariable logistic model and a combination of genetic, phenotypic and interaction variables is an effective approach for stratifying women in the Spanish population according to individual risk of suffering breast cancer within a 5-year period, with a capacity similar to that observed in other studies in European and non-European populations. Due to the nature of our study, different biases could have affected the precision of the results; for example, there may have been selection and length biases. Additionally, the small size of our cohort could have led to overfitting of the model in terms of risk estimation or the over/under representation of a specific tumor type. However, in spite of these limitations, our analysis provides proof of concept in a population that has not been studied until now. Larger series are necessary in order to confirm our data and initiate the use of this type of screening method in the Spanish population.

## Conclusions

Our results endorse the capacity of the multivariable model to stratify women according to their risk of developing breast cancer. Some bias could be present in the study and could have affected the precision of our results; however, the analysis provides proof of concept in a poorly studied population and opens up the possibility of its use in the routine screening of the Spanish population.

## Supplementary Information

**Additional file 1.**

## Data Availability

The datasets used and/or analysed during the current study are available from the corresponding author on reasonable request.

## Abbreviations

CNIO: Spanish National Cancer Center
SNP: Single-nucleotide polymorphism
OR: Odd ratio
GWAS: Genome-wide association studies
PRS: Polygenic risk score
MD: Mammographic density
COGS: European Collaborative Oncological Gene Environment Study
DNA: Deoxyribonucleic acid
LRT: Likelihood ratio test
AUC: Area under the curve
CI: Confidence interval
SD: Standard deviation
