# Supplementary material for: Combination of phenotype and polygenic risk score in breast cancer risk evaluation in the Spanish population: a case –control study
Source: BMC Cancer. 2020 Nov 10;20:1079. doi: 10.1186/s12885-020-07584-9 (PMC7654173; doi:10.1186/s12885-020-07584-9)
Supplement: Supplementary file 1 — Additional file 1. [file 12885_2020_7584_MOESM1_ESM.pdf]

Supplementary Table S1.

	SNPs	MAF	Alleles	OR
1	rs10069690	0.276	C/T	1.06
2	rs1045485	0.15	G/C	0.88
3	rs10472076	0.36	T/C	1.03
4	rs10474352	0.159	C/T	0.94
5	rs10771399	0.145	A/G	0.79
6	rs10816625	0.103	A/G	1.11
7	rs10931936	0.224	C/T	0.88
8	rs10941679	0.248	A/G	1.15
9	rs10995190	0.192	G/A	0.86
10	rs10995201	0.187	A/G	0.9
11	rs11199914	0.299	C/T	0.96
12	rs11249433	0.5	A/G	1.11
13	rs113577745	0.093	C/G	1.08
14	rs11552449	0.21	C/T	1.04
15	rs11571833	0.014	A/T	1.35
16	rs11814448	0.028	A/C	1.12
17	rs11820646	0.402	C/T	0.96
18	rs11977670	0.355	G/A	1.06
19	rs12422552	0.313	G/C	1.06
20	rs12443621	0.504	A/G	1.11
21	rs12710696	0.308	C/T	1.03
22	rs1292011	0.416	A/G	0.92
23	rs13066793	0.107	A/G	0.94
24	rs13281615	0.486	A/G	1.11
25	rs13294895	0.178	C/T	1.06
26	rs13329835	0.178	A/G	1.07
27	rs13365225	0.145	A/G	0.91
28	rs13387042	0.537	G/A	1.2
29	rs1353747	0.075	T/G	0.96
30	rs1432679	0.509	T/C	1.08
31	rs1550623	0.187	A/G	0.94
32	rs16857609	0.248	C/T	1.06
33	rs16991615	0.079	G/A	1.1
34	rs17356907	0.285	A/G	0.91
35	rs17817449	0.374	T/G	0.95
36	rs1895062	0.481	A/G	0.94
37	rs204247	0.444	A/G	1.04
38	rs2046210	0.397	G/A	1.11
39	rs2236007	0.252	G/A	0.93
40	rs2290203	0.215	G/A	0.94
41	rs2363956	0.463	G/T	1.19

42	rs2588809	0.243	C/T	1.06
43	rs2747652	0.477	C/T	0.94
44	rs2823093	0.243	G/A	0.94
45	rs2943559	0.093	A/G	1.1
46	rs2981579	0.397	G/A	1.43
47	rs2992756	0.518	C/T	1.06
48	rs3757322	0.369	T/G	1.08
49	rs3760982	0.495	G/A	1.05
50	rs3803662	0.252	G/A	1.2
51	rs3903072	0.435	G/T	0.97
52	rs4442975	0.481	G/T	0.89
53	rs45631563	0.042	A/T	0.81
54	rs4784227	0.238	C/T	1.23
55	rs4808801	0.336	A/G)	0.93
56	rs4849887	0.15	C/T	0.91
57	rs4973768	0.551	C/T	1.11
58	rs554219	0.141	C/G	1.21
59	rs58058861	0.117	G/A	1.06
60	rs58847541	0.159	G/A	1.08
61	rs6001930	0.103	T/C	1.12
62	rs614367	0.146	C/T	1.15
63	rs616488	0.322	A/G	0.94
64	rs6472903	0.145	T/G	0.94
65	rs6504950	0.252	G/A	0.95
66	rs6507583	0.084	A/G	0.92
67	rs6762644	0.313	A/G	1.05
68	rs6815814	0.444	A/C	1.06
69	rs6828523	0.084	C/A	0.91
70	rs704010	0.453	C/T	1.07
71	rs7072776	0.308	G/A	1.05
72	rs720475	0.304	G/A	0.96
73	rs72749841	0.243	T/C	0.93
74	rs72755295	0.028	A/G	1.15
75	rs72826962	0.014	C/T	1.2
76	rs7297051	0.234	C/T	0.89
77	rs73161324	0.033	C/T	1.06
78	rs7529522	0.313	T/C	1.06
79	rs75915166	0.061	C/A	1.28
80	rs78269692	0.056	T/C	1.09
81	rs8009944	0.262	A/C	0.88
82	rs8170	0.14	G/A	1.26
83	rs865686	0.388	T/G	0.89
84	rs889312	0.248	A/C	1.13

85	rs909116	0.486	C/T	1.17
86	rs9397437	0.089	G/A	1.17
87	rs941764	0.383	A/G	1.03
88	rs9693444	0.364	C/A	1.06
89	rs9790517	0.192	C/T	1.04
90	rs9790879	0.388	T/C	1.1
91	rs9833888	0.215	G/T	1.06
92	rs527616	0.369	G/C	0.97
